# Plant‐based dietary patterns and cognitive function: A prospective cohort analysis of elderly individuals in China (2008–2018)

**DOI:** 10.1002/brb3.2670

**Published:** 2022-07-14

**Authors:** Anna Zhu, Changzheng Yuan, Jules Pretty, John S. Ji

**Affiliations:** ^1^ Division of Clinical Epidemiology and Aging Research German Cancer Research Center (DKFZ) Heidelberg Germany; ^2^ School of Public Health Zhejiang University Zhejiang China; ^3^ Harvard T.H. Chan School of Public Health Harvard University Boston Massachusetts USA; ^4^ School of Life Sciences University of Essex Colchester UK; ^5^ Vanke School of Public Health Tsinghua University Beijing China

**Keywords:** cognitive function, healthy longevity, plant‐based dietary patterns

## Abstract

**Introduction:**

Plant‐based diets confer health benefits, especially on the prevention of noncommunicable diseases. The relationship between plant‐based dietary patterns on cognitive function as a neurological outcome needs more evidence. We aimed to assess the associations between plant‐based dietary patterns and cognitive function among Chinese older adults.

**Methods:**

We used four waves (2008–2018) of the Chinese Longitudinal Healthy Longevity Survey. We included 6136 participants aged 65 years and older with normal cognition at baseline. We constructed an overall plant‐based diet index (PDI), healthful plant‐based diet index (hPDI), and unhealthful plant‐based diet index (uPDI) from questionnaires. We used the Mini‐Mental State Examination (MMSE) to assess cognitive function. We used the multivariable‐adjusted generalized estimating equation to explore the corresponding associations.

**Results:**

The multivariable‐adjusted models showed inverse associations between plant‐based dietary patterns and cognitive function. The highest quartiles of PDI and hPDI were associated with a 55% (odds ratio [OR] = 0.45, 95% CI: 0.39, 0.52) decrease and a 39% (OR = 0.61, 95% CI: 0.54, 0.70) decrease in the odds of cognitive impairment (MMSE < 24), compared with the lowest quartile. In contrast, the highest quartile of uPDI was associated with an increased risk (OR = 2.03, 95% CI: 1.79, 2.31) of cognitive impairment. We did not observe pronounced differences by selected socioeconomic status, physical activity, residential greenness, and APOE ε4 status.

**Conclusions:**

Our findings suggested that adherence to healthy plant‐based dietary patterns was associated with lower risks of cognitive impairment among older adults, and unhealthy plant‐based dietary patterns were related to higher risks of cognitive impairment.

## Graphical Abstract

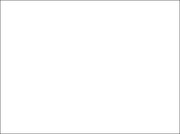

## 1 INTRODUCTION

In 2016, there were 43.8 million cases of dementia, which was the fifth leading cause of death globally (GBD 2016 Dementia Collaborators, 2019). There is no cure for dementia and Alzheimer's disease, as the current therapeutic pathways merely alleviate symptoms or slow the progressions (Livingston et al., 2020). Nutrition, along with modifiable behavioral and environmental factors, maybe a prudent public health intervention to prevent or delay the onset of dementia (Erickson et al., 2011; Livingston et al., 2020; Scarmeas et al., 2018). Evidence has showed potential benefits of certain nutrients, food groups, and dietary patterns on cognitive outcomes (Scarmeas et al., 2018; Smith & Blumenthal, 2016; Tucker, 2016). These studies served as evidence for adoption of the Mediterranean, and Dietary Approaches to Stop Hypertension (DASH) (Lourida et al., 2013; Scarmeas et al., 2018; Singh et al., 2014). These dietary patterns suggest abundance intake of fruits, vegetables, and whole grains, with limited intake of animal foods and processed foods.

There is emerging evidence of plant‐based dietary patterns conferring benefits for neurological health (Medawar et al., 2019; Rajaram et al., 2019). The Singapore Chinese Health Study of 16,948 men and women reported that plant‐based dietary patterns in middle life were associated with lower risks of cognitive impairment in late life (Wu et al., 2019). Another cohort of 12,062 participants from Taiwan found that vegetarians had a 38% lower risk of dementia, compared with nonvegetarians (Lin et al., 2019). A study of Japanese elderly discovered diets with plentiful plant foods and fish were associated with better cognitive function (Okubo et al., 2017). However, not all findings yielded significant results. A cohort of 13,588 healthy adults in the United States showed that changes in cognitive function and risks of developing dementia in later life did not differ by the meat or plant‐based dietary patterns (Dearborn‐Tomazos et al., 2019).

China and other developing countries in Asia traditionally consume a large amount of plant food, while simultaneously transitioning to consuming more animal‐based foods with rising income (Bishwajit et al., 2014). We need to better understand dietary transition during economic developments and relationships between plant‐based dietary patterns and cognitive function. Our study aimed to explore plant‐based dietary patterns and their relationships with cognitive function, by using the Chinese Longitudinal Healthy Longevity Survey (CLHLS).

## 2 MATERIALS AND METHODS

### 2.1 Study population

Started in 1998, the CLHLS aimed to study the determinants of healthy longevity. The CLHLS has a nationally representative sample, with participants recruited from 22 out of 31 provinces in China. The CLHLS applied a multistage, stratified cluster sampling in 631 randomly selected cities and counties where the Han Chinese are the largest majority. A more detailed description of the sampling design can be found elsewhere (Zeng et al., 2008).

Our study used 2008, 2011, 2014, and 2018 survey data from the CLHLS. To avoid reverse causation and to reduce recall bias caused by cognitive impairment, we restricted the analysis to 6136 participants with follow‐up surveys and normal cognitive function (baseline MMSE score ≥ 24) at baseline. More details about participant inclusion can be found in Figure S1. A comparison of baseline characteristics of those with follow‐up surveys and those without follow‐up surveys can be found in Table S1.

### 2.2 Dietary assessment

We evaluated plant‐based dietary patterns by constructing the overall plant‐based diet index (PDI), healthful plant‐based diet index (hPDI), and unhealthful plant‐based diet index (uPDI), the adapted approach used by Satija et al. (2016). We included 16 food groups, and categorized them into three groups, including animal food groups and healthy and less healthy plant food groups, which were differently associated with a set of health outcomes (Anastasiou et al., 2017; Dong et al., 2016). We scored the indices according to intake frequency. Although servings or quintiles of intake are commonly used, using a nonquantitative food frequency questionnaire to assess dietary patterns has been demonstrated to be reliable and valid in some studies (Mohammadifard et al., 2015; Saeedi et al., 2016; Wong et al., 2012). We gave positive scores to higher intake frequency of plant food groups, and reverse scores to higher intake frequency of animal food groups when coding PDI. Similarly, we gave positive scores to healthy plant food groups and reverse scores to less healthy plant food groups and animal food groups for hPDI, and positive scores to less healthy plant food groups and reverse scores to healthy plant and animal food groups for uPDI. More details on constructing and scoring PDI, hPDI, and uPDI could be found in Table S2 and Methods in the Supporting Information.

### 2.3 Cognitive function assessment

We used the adapted Chinese version of the Mini‐Mental State Examination (MMSE) (score range 0–30) to measure cognitive function (Zhu et al., 2019), which has been validated for reliability in prior findings (Zeng et al., 2017, 2008; Zhu et al., 2019). MMSE assesses cognitive function in five dimensions: orientation, registration, attention and calculation, recall, and language (Folstein et al., 1975). Higher scores indicated better cognitive function (Li et al., 2017; Tombaugh & McIntyre, 1992). We dichotomized MMSE scores to ≥24 as normal cognition and <24 as cognitive impairment in the main analysis, which has been widely used as a cutoff point to indicate cognitive impairment (An et al., 2018; Chong et al., 2019; Obbels et al., 2019; Tombaugh & McIntyre, 1992). We also conducted a sensitivity analysis by using the MMSE score of 18 as the cutoff point (Matusik et al., 2012; Tombaugh & McIntyre, 1992).

### 2.4 Covariates

We assessed demographic characteristics, socioeconomic status, health behavior, and health status. The covariates included age, sex, urban/rural residence, education (with or without formal education), physical activity (yes or no), and geographic regions (Central, Eastern, Northeastern, Northern, Northwestern, Southern, and Southwestern China). We dichotomized main occupation to professional work (professional and technical personnel, government, and management) and nonprofessional work (agriculture, fishing, service, industry, and housework). We categorized financial status to financial independence, where participants were financially independent with their work and retirement wage, and financial dependence, where participants financially relied on other family members. We calculated the social and leisure activity index by considering seven activities: gardening, personal outdoor activities excluding exercise, raising poultry or pets, reading, playing cards or mahjong, listening to the radio or watching TV, and participating in organized social activities (Zeng et al., 2010). Each activity was scored 0 (no) or 1 (yes), and the index ranged from 0 to 7. We also evaluated smoking and drinking status (never, former, and current), body mass index (BMI), vitamin A/C/E intake (almost every day, ≥1 time per week, ≥1 time per month, occasionally, rarely, or never), status of hypertension, diabetes, heart disease, cerebrovascular disease, and dyslipidemia (yes, no, and unknown). These diseases were potential risk factors of cognitive decline (Baumgart et al., 2015). We additionally considered residential green space in the stratified analysis because green space might influence vegetable consumption (Yuen et al., 2019). Based on residential addresses of participants, we calculated normalized difference vegetation index from Moderate‐Resolution Imaging Spectro‐Radiometer in the National Aeronautics and Space Administration's Terra Satellite, to indicate levels of green space. More details for measurements of residential green space could be found elsewhere (Ji et al., 2019).

### 2.5 Statistical analysis

Generalized estimating equation (GEE) applies a population‐level model based on a quasi‐likelihood function, and estimates the population‐averaged estimates of the response to changes in covariates (Ballinger, 2004; Wang, 2014). GEE has been widely used in longitudinal analysis with response variables that were not normally distributed. We used the GEE to calculate the odds ratio (OR) and 95% confidence interval (CI) for associations between plant‐based dietary patterns and cognitive impairment. Both the plant‐based dietary patterns and cognitive function were time varying and were repeatedly measured in 2008, 2011, 2014, and 2018. We further adjusted the regression models for time‐varying health behavior and health status in the sensitivity analysis. In addition, because education attainment is an important factor of MMSE score, we applied education‐specific cutoff points to categorize MMSE score as a sensitivity analysis (Wu et al., 2019). We used the MMSE score of 18, 20, and 24 as the cutoff points for the participants without formal education, primary school education (1–6 years), and secondary school or higher education (>6 years), respectively. Furthermore, we conducted an additional longitudinal analysis, regardless of participants’ cognitive function at baseline, as supplementary analysis. We implemented correlation analysis among different food groups. We also conducted food component analysis to examine the individual contribution and the contribution of healthy plant food, less healthy plant food, and animal food groups. Several studies indicated that APOE ε4 status is a risk factor of Alzheimer's disease, and may modify the associations (Morris et al., 2015; Olsson et al., 2015). Thus, we conducted stratified analysis by APOE ε4 status. We presented the results of three regression models adjusted for different covariates. Model 1 was adjusted for age. Model 2 was multivariable adjusted for age, sex, marital status, urban/rural residence, education, occupation before age 60, financial status, social and leisure activity, smoking and drinking status, physical activity, and geographic regions. Model 3 was additionally adjusted for BMI, vitamin A/C/E intake, and status of five cardiometabolic diseases, including hypertension, diabetes, heart disease, cerebrovascular disease, and dyslipidemia. The participants with normal cognition (MMSE ≥ 24) were considered as the reference groups. We plotted the restricted cubic splines to explore the dose–response relationship. All statistical analysis was conducted by using STATA 14.0. Statistical significance was defined by *p* < .05 in two‐sided testing.

## 3 RESULTS

We presented the baseline characteristics among 6136 participants with follow‐up surveys and normal cognition at baseline in Table 1. Their mean age was 80 (standard deviation [SD] = 9.83) years old, 53.67% were males, and 82.58% were rural residents.

**TABLE 1:** Baseline characteristics by quartiles of PDI, among the participants with follow‐up surveys and normal cognition at baseline (*n* = 6136)

Characteristics	Quartile 1	Quartile 2	Quartile 3	Quartile 4	Total (*n*, %)
MMSE score^a^	28.37 ± 1.50	28.39 ± 1.57	28.50 ± 1.52	28.48 ± 1.51	28.43 ± 1.53
Age (years)^a^	81.54 ± 10.20	79.98 ± 9.80	78.83 ± 9.59	77.32 ± 9.14	79.53 ± 9.83
Sex (% males)	778 (50.29)	953 (53.36)	836 (55.15)	726 (56.41)	3293 (53.67)
Married (%)	649 (41.95)	903 (50.56)	821 (54.16)	735 (57.11)	3108 (50.65)
Rural residents (%)	1353 (87.46)	1445 (80.91)	1209 (79.75)	1060 (82.36)	5067 (82.58)
No formal education (%)	784 (50.68)	856 (47.93)	665 (43.87)	558 (43.36)	2863 (46.66)
Nonprofessional work (%)	1438 (92.95)	1607 (89.98)	1345 (88.72)	1139 (88.50)	5529 (90.11)
Financial dependence (%)	1067 (68.97)	1067 (59.74)	862 (56.86)	678 (52.68)	3674 (59.88)
Social and leisure activity index^a^	2.66 ± 1.40	2.81 ± 1.43	2.96 ± 1.45	3.14 ± 1.45	2.88 ± 1.44
Current smoker (%)	290 (18.75)	391 (21.89)	377 (24.87)	363 (28.21)	1421 (23.16)
Current drinker (%)	295 (19.07)	370 (20.72)	328 (21.64)	321 (24.94)	1314 (21.41)
Physical activity (%)	485 (31.35)	671 (37.57)	587 (38.72)	492 (38.23)	2235 (36.42)
Geographic region					
Central China (%)	180 (11.64)	402 (22.51)	296 (19.53)	260 (20.20)	1138 (18.55)
Eastern China (%)	285 (18.42)	591 (33.09)	683 (45.05)	634 (49.26)	2193 (35.74)
Northeastern China (%)	71 (4.59)	136 (7.61)	119 (7.85)	114 (8.86)	440 (7.17)
Northern China (%)	28 (1.81)	89 (4.98)	72 (4.75)	72 (5.59)	261 (4.25)
Northwestern China (%)	6 (0.39)	20 (1.12)	20 (1.32)	27 (2.10)	73 (1.19)
Southern China (%)	807 (52.17)	393 (22.00)	182 (12.01)	60 (4.66)	1442 (23.50)
Southwestern China (%)	170 (10.99)	155 (8.68)	144 (9.50)	120 (9.32)	589 (9.60)
BMI (kg/m^2^)^a^	20.37 ± 3.55	21.57 ± 11.14	21.47 ± 3.49	21.74 ± 3.81	21.28 ± 6.75
Daily vitamin A/C/E intake	71 (4.59)	117 (6.55)	96 (6.33)	101 (7.85)	385 (6.27)
Self‐reported hypertension	319 (20.62)	410 (22.96)	392 (25.86)	286 (22.22)	1407 (22.93)
Self‐reported diabetes	38 (2.46)	68 (3.81)	57 (3.76)	31 (2.41)	194 (3.16)
Self‐reported heart diseases	120 (7.76)	201 (11.25)	174 (11.48)	122 (9.48)	617 (10.06)
Self‐reported cerebrovascular disease	67 (4.33)	116 (6.49)	94 (6.2)	58 (4.51)	335 (5.46)
Self‐reported dyslipidemia	23 (1.49)	33 (1.85)	33 (2.18)	23 (1.79)	112 (1.83)

^a^ Mean ± SD was reported.

**FIGURE 1:**
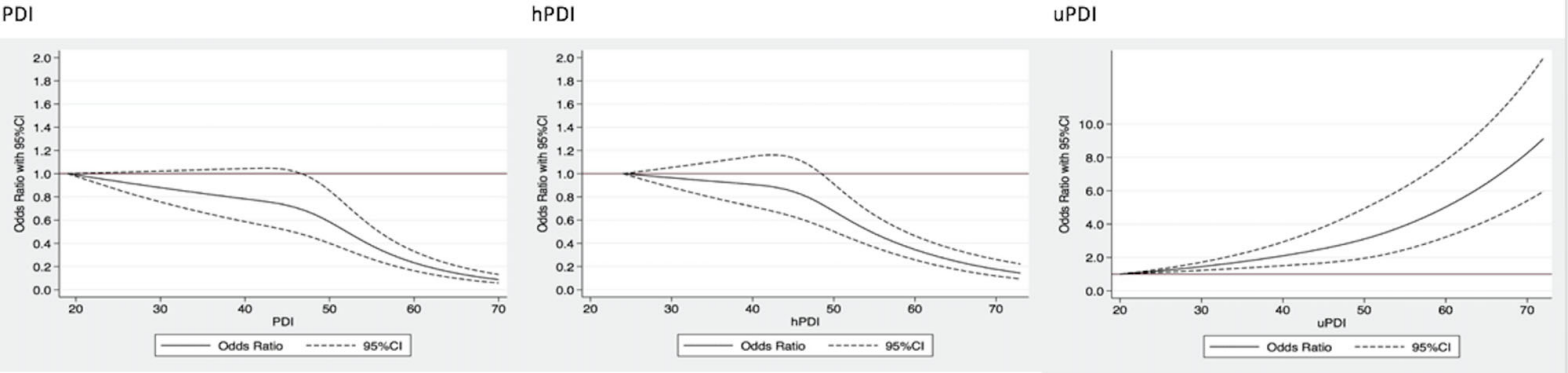
Curves for plant‐based diets and development of cognitive impairment among the participants with follow‐up surveys and with normal cognitive function at baseline

Table 2 showed the risk of developing cognitive impairment by quartiles of plant‐based diet indices. In Model 2, the highest quartile of PDI and hPDI was significantly associated with a 55% (OR = 0.45, 95% CI: 0.39, 0.52) decrease and a 39% (OR = 0.61, 95% CI: 0.54, 0.70) decrease in risk of developing cognitive impairment, respectively. In contrast, the highest quartile of uPDI was significantly associated with an OR of 2.03 (95% CI: 1.79, 2.31) of cognitive impairment. After additional adjustment for BMI, vitamin A/C/E intake, and status of five cardiometabolic diseases (Model 3), the associations remained significant and the effect estimates were similar. These findings were consistent with the dose–response curves (shown in Figure 1). The results of the main analysis were also consistent with the results of sensitivity analysis by using education‐specific cutoff points for MMSE categorization (shown in Table S3), and by adjusting for time‐varying health behavior and health status (shown in Table S4). Additionally, the associations were stronger than the analysis without excluding the participants with cognitive impairment at baseline (PDI: OR = 0.69 [95% CI: 0.63, 0.76]; hPDI: OR = 0.78 [95% CI: 0.71, 0.86]; uPDI: OR = 1.93 [95% CI: 1.75, 2.12]) (shown in Table S5).

**TABLE 2:** Odds ratios (95% CI) of developing cognitive impairment by quartiles of plant‐based diet indices among the participants with follow‐up surveys, and with normal cognition at baseline (*n* = 6136)

Diet indices	*N* (median)^a^	MMSE (mean)^a^	Model 1		Model 2		Model 3	
			OR (95% CI)	*p*	OR (95% CI)	*p*	OR (95% CI)	*p*
PDI								
Quartile 1	1547 (42)	28.37	Reference	Reference	Reference	Reference	Reference	Reference
Quartile 2	1786 (48)	28.39	0.91 (0.82, 1.00)	.050	0.90 (0.81, 1.00)	.058	0.90 (0.81, 1.01)	.064
Quartile 3	1516 (52)	28.49	0.64 (0.57, 0.71)	<.001	0.64 (0.57, 0.72)	<.001	0.64 (0.57, 0.72)	<.001
Quartile 4	1287 (57)	28.47	0.43 (0.38, 0.49)	<.001	0.45 (0.39, 0.51)	<.001	0.45 (0.39, 0.52)	<.001
*p*‐value for trend			–	<.001	–	<.001	–	<.001
hPDI								
Quartile 1	1536 (40)	28.36	Reference	Reference	Reference	Reference	Reference	Reference
Quartile 2	1603 (46)	28.34	0.87 (0.79, 0.96)	.006	0.90 (0.81, 1.00)	.042	0.90 (0.81, 1.00)	.044
Quartile 3	1745 (49)	28.52	0.71 (0.64, 0.80)	<.001	0.76 (0.67, 0.85)	<.001	0.76 (0.67, 0.85)	<.001
Quartile 4	1252 (54)	28.51	0.56 (0.50, 0.63)	<.001	0.61 (0.54, 0.69)	<.001	0.61 (0.54, 0.70)	<.001
*p*‐value for trend			–	<.001	–	<.001	–	<.001
uPDI								
Quartile 1	1769 (43)	28.73	Reference	Reference	Reference	Reference	Reference	Reference
Quartile 2	1418 (49)	28.46	1.32 (1.17, 1.49)	<.001	1.18 (1.04, 1.34)	.009	1.17 (1.03, 1.33)	.014
Quartile 3	1723 (53)	28.31	1.74 (1.56, 1.95)	<.001	1.48 (1.31, 1.67)	<.001	1.47 (1.30, 1.66)	<.001
Quartile 4	1226 (58)	28.15	2.56 (2.28, 2.88)	<.001	2.05 (1.81, 2.33)	<.001	2.03 (1.79, 2.31)	<.001
*p*‐value for trend			–	<.001	–	<.001	–	<.001

Abbreviations: hPDI, healthful plant‐based diet index; PDI, overall plant‐based diet index; uPDI, unhealthful plant‐based diet index.

^a^ Because MMSE, PDI, hPDI, and uPDI were multiply measurements, descriptive statistics at baseline were reported. Model 1 was adjusted for age. Model 2 was multivariable adjusted for age (years), sex (male or female), marital status (married or unmarried), urban/rural residence, education (with or without formal education), occupation before age 60 (professional or nonprofessional work), financial status (financial independence or dependence), social and leisure activity, smoking and drinking status (never, former, or current smokers/drinkers), physical activity (yes or no), and geographic regions (Central China, Eastern China, Northeastern China, Northern China, Northwestern China, Southern China, and Southwestern China). Model 3 was additionally adjusted for BMI (<18.5, 18.5–25.0, or ≥25.0 kg/m^2^), vitamin A/C/E intake (almost everyday, ≥1 time/week, ≥1 time/month, occasionally, rarely, or never), and status of five cardiometabolic diseases, including hypertension, diabetes, heart disease, cerebrovascular disease, and dyslipidemia (yes, no, or unknown).

**FIGURE 2:**
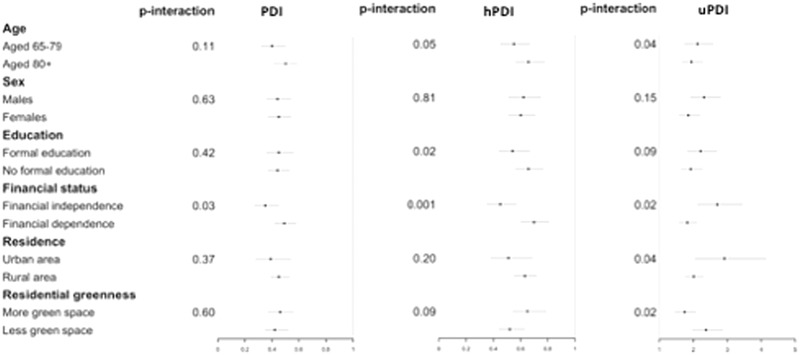
Stratified analysis for the odds ratios of highest quartile of plant‐based dietary indices on cognitive impairment among the participants with follow‐up surveys and with normal cognitive function at baseline

Figure 2 reported the stratified analysis of the associations. The effects of plant‐based dietary patterns on cognitive functions were slightly stronger among the participants who were younger, males, urban residents, with formal education, financially independent, and living in areas with less green space. There were significant interactions between plant‐based dietary patterns and financial status. Overall, we did not observe pronounced differences by other characteristics. A proportion of participants had information on APOE ε4 status, who were mainly recruited from study sites of longevity. In this subgroup, we further stratified the analysis by APOE ε4 status (shown in Table S6). Although the differences by APOE ε4 status were nonsignificant, we observed slightly stronger positive effects of PDI and hPDI, but stronger negative effects of uPDI on cognitive impairment among APOE ε4 carriers than non‐ε4 carriers (PDI: OR = 0.44 [95% CI: 0.30, 0.66] vs. OR = 0.41 [95% CI: 0.34, 0.51]; hPDI: OR = 0.68 [95% CI: 0.47, 1.00] vs. OR = 0.60 [95% CI: 0.50, 0.73]; uPDI: OR = 2.11 [95% CI: 1.45, 3.05] vs. OR = 2.06 [95% CI: 1.71, 2.47]).

We constructed a modified hPDI with positively scoring fish intake, which was shown to benefit cognitive function, and tested its effects on cognitive function (see Table S7). Compared to hPDI, the modified hPDI showed stronger effects on cognitive impairment (OR = 0.52, 95% CI: 0.46, 0.59). Additionally, we used MMSE score of 18 to compare the sensitivity. We found that the associations were still significant and became slightly stronger (PDI: OR = 0.32 [95% CI: 0.26, 0.39]; hPDI: OR = 0.47 [95% CI: 0.39, 0.56]; uPDI: OR = 2.39 [95% CI: 2.00, 2.86]) (shown in Table S8).

The correlation coefficients among different food groups range from −0.22 to 0.39 (shown in Figure S2). To test the contribution of individual food components and food groups, we included all food groups simultaneously in the multivariable‐adjusted models (shown in Tables S9 andS10). We found that more frequent consumption of several healthy plant food inclusive of fruit and fresh vegetables and fish was significantly associated with lower risks of cognitive impairment; positive associations were observed among vegetable oil, meat, and dairy products (shown in Table S9). Additionally, we discovered that more frequent intake of healthy plant food was associated with lower risks of cognitive impairment (OR = 0.34, 95% CI: 0.29, 0.40). No significant association was found for less healthy plant food (OR = 0.93, 95% CI: 0.81, 1.06). There was a weak inverse association for animal food (OR = 0.87, 95% CI: 0.77, 0.99) (shown in TableS10).

## 4 DISCUSSION

In this prospective cohort study of 6136 Chinese older adults with normal cognition at baseline, we found an inverse association of plant‐based dietary patterns and risks of cognitive impairment. The associations were consistent across subgroups of age, sex, SES, and APOE ε4 status. The observed associations were slightly stronger among participants with higher SES, which is closely associated with favorable dietary patterns and more vegetable and fruit intake (Czarnocinska et al., 2020; Desbouys et al., 2020). Slightly stronger associations were also observed among APOE ε4 carriers, which was consistent with other studies. APOE ε4 carriers were shown to be more vulnerable to poor cognitive function caused by environmental determinants, like air pollution and dietary patterns (Kulick et al., 2020; Prinelli et al., 2019). Our finding indicates the health benefits of plant‐based dietary patterns on cognitive function among older adults, and may provide evidence for clinical intervention for dementia prevention.

Our findings were consistent with several studies. The Singapore Chinese Health Study showed that the highest level of PDI and hPDI was associated with an 18% (OR = 0.82, 95% CI: 0.71, 0.94) decrease and a 22% (OR = 0.78, 95% CI: 0.68, 0.90) decrease in risks of cognitive impairment (Wu et al., 2019). Our findings reported stronger effect estimates than the Singapore study, and the effect estimates of PDI on cognitive impairment were stronger than hPDI. This may be due to our study population being in a developing country, and among a select group of elderly individuals. Furthermore, the individual component analysis found that frequent intake of salt‐preserved vegetables was associated with lower risks of cognitive impairment. Salt‐preserved vegetables were common in diets in Asia, with sodium intake linked to the development of coronary heart diseases. However, its associations with cognitive function were not in agreement among several studies (Chen et al., 2017; Huang et al., 2021; Okubo et al., 2017; Xu et al., 2018). This could be because salt‐preserved vegetables include micronutrients that may benefit cognition. As an example, *kimchi* (Korean traditional pickled cabbage) contains vitamin B12 and vitamin E, which were associated with better cognitive function (Chen et al., 2017), although the biological mechanisms need further investigation. In our analysis, the effect estimates on cognitive impairment of salt‐preserved vegetables were shown to be beneficial, compared to sugar (OR for almost every day = 0.74 [95% CI: 0.65, 0.85] vs. OR = 1.04 [95% CI: 0.91, 1.19]), probably attenuating some effect estimates. This might partly explain why the overall plant‐based dietary pattern had stronger effects on cognitive function than the healthful plant‐based dietary pattern.

Inverse associations between plant‐based dietary patterns and cognitive function were reported in some (Lin et al., 2019; Okubo et al., 2017) but not in all studies, depending on the length of follow‐up (Akbaraly et al., 2019; Medawar et al., 2019). For instance, a US cohort reported that neither the diet with high consumption of meats (OR = 1.06, 95% CI: 0.92, 1.22) nor the diet with high intake of fruits and vegetables at middle age (OR = 0.99, 95% CI: 0.88, 1.12) was associated with incident dementia in later life after a 20‐year follow‐up (Dearborn‐Tomazos et al., 2019). The Whitehall II study with a median follow‐up of 25 years showed that dietary patterns at midlife were not significantly associated with subsequently increased risks of dementia. Both have more than 20 years of follow‐up, much more than other studies that reported inverse associations. The inconsistency may be partly explained by duration of follow‐up. Dementia has a progressive neurodegenerative nature with pathophysiological changes that occur over 15–20 years. In the meanwhile, effects of changes in dietary patterns may be neglected (Akbaraly et al., 2019).

In addition, our study observed stronger effects of modified hPDI than hPDI, indicating that plant‐based dietary patterns including some healthy animal food like fish have more benefits on cognitive function. In the individual food group analysis, although we did not find significant associations between less healthy plant food and cognitive function, we could not rule out possible negative effects caused by other less healthy plant food. Since we only considered refined grains, salt‐preserved vegetables, and sugar, we may have missed other less healthy food like sweetened beverages, which have been found to be associated with cognitive impairment (Muñoz‐García et al., 2020).

There are several mechanisms for benefits of plant‐based dietary patterns on cognitive function. Primarily, adherence to healthy diets was associated with better weight control and lower risks of cardiometabolic diseases (AlEssa et al., 2017), which were risk factors of cognitive impairment (Lyall et al., 2017). In our study, adjustment for BMI and cardiometabolic diseases did not alter the result, which may be due to underreported cardiometabolic diseases. In addition, vegetables, fruits, and vegetable oil have rich nutrients including polyphenols, unsaturated fatty acids like omega‐3 and ‐6, and dietary fiber, which have been shown to reduce inflammation and oxidative stress (Calder, 2015; Duvall & Levy, 2016; Kwon, 2020; Ricker & Haas, 2017; Zhang & Tsao, 2016), ultimately influencing the pathogenesis of neurodegenerative disorder (Liu et al., 2017; Marsland et al., 2015). There were consistent inverse associations between dietary flavonoids (the family of polyphenolic compounds) intake and cognitive function (Godos, Caraci, et al., 2020; Kesse‐Guyot et al., 2012; Letenneur et al., 2007; Root et al., 2015; Shishtar et al., 2020). Flavonoids could modulate systemic inflammation and oxidative stress and affect metabolites linked to the gut–microbiome–brain axis (Godos, Caraci, et al., 2020). Unsaturated fatty acids were involved in modulating metabolic, immune, and inflammatory processes in the central nervous system (Godos, Currenti, et al., 2020). Furthermore, some evidence showed that healthful plant‐based dietary patterns could benefit gastrointestinal microbiome, directly affecting neurotransmitters and also acting as a part of gut–brain axis (Godos, Currenti, et al., 2020). Gastrointestinal microbiome further promoted the metabolism of fiber and polyphenols, and inhibited the metabolism of bile acids, choline and L‐carnitine, and amino acid. These processes could affect the central nervous system as well (Collins et al., 2012; Glick‐Bauer & Yeh, 2014; Jacka, 2017). Other nutrients like B‐group vitamins and amino acids also played roles in the central nervous system (Godos, Currenti, et al., 2020).

The strengths of our study included using a longitudinal analysis with a 10‐year follow‐up, multiply measurements of dietary patterns and cognitive function, a national representative sample. We also carefully adjusted for lots of confounding variables, yielding relatively valid estimates for the association of interest. Additionally, we conducted several sensitivity analyses, like using different cutoff points for cognitive impairment, which reported consistent findings with the main analysis. We also tested whether APOE ε4 carriers modified the associations. Furthermore, our main analysis only included the participants with normal cognition at baseline to avoid reverse causality. Our study generated evidence from East Asia to a Western‐centric nutrition research paradigm.

Our study also had several limitations. First, our inclusion and exclusion of participants might cause selection bias and reduce generalizability. We excluded the participants who died or were lost to follow‐up before the second survey, reducing around 40% of the sample size due to advanced age at recruitment (mean: 87 years old). To consider the selection effect, compared to the participants with follow‐up surveys, those without follow‐up were much younger (82 vs. 92 years old). They also tended to have lower PDI and hPDI but higher uPDI (shown in TableS1). We also excluded the participants with cognitive impairment at baseline to reduce recall bias. Second, there may be residual confounding. Cardiometabolic diseases were self‐reported, unverified by the clinical diagnosis, and underreported. But it shall not bias our results since they were not key covariates. Third, we used intake frequency to score the diets, rather than using servings or quintiles of intake per day. We do not have information on portion sizes, but frequency of intake may be more important to distinguish between high and low consumption of fruits and vegetables (63). Several studies have demonstrated the reliability and validity of using nonquantitative food frequency questionnaires to assess dietary patterns (Mohammadifard et al., 2015; Saeedi et al., 2016; Wong et al., 2012). A further complication is that our food questionnaire may not be standardized enough to be comparable with other nutrition studies, and face challenges translating into nutrition recommendations. Lastly, we used MMSE rather than the clinical diagnosis. Different versions, scoring, and interpretation may also cause some inconsistencies (Tombaugh & McIntyre, 1992), although the adapted Chinese version used in our study was demonstrated reliable and valid in prior research (Zeng et al., 2008).

## 5 CONCLUSIONS

We found that higher adherence to plant‐based dietary patterns was significantly associated with lower risks of cognitive impairment among older adults. Our findings suggest that maintaining a diet rich in healthy plant foods with some healthy animal foods like fish could benefit cognitive function among older adults and may contribute to the prevention of onset and delay of cognitive impairment. On a population level, wider adoption of plant‐based dietary patterns may contribute to reducing incidences of cognitive impairment and dementia in the elderly. Given the greenhouse gas emission of meat production, higher plant‐based diets can also contribute to climate change mitigation.

## CONFLICT OF INTEREST

The authors declare no conflict of interest.

## AUTHOR CONTRIBUTIONS

AZ and JSJ conceived and designed the study design. AZ and JSJ conducted statistical analysis. AZ and JSJ drafted the manuscript. CY and PJ helped interpret data. All authors contributed to the interpretation of findings, provided revisions to the manuscript, and approved the final manuscript.

### PEER REVIEW

The peer review history for this article is available at https://publons.com/publon/10.1002/brb3.2670

## Supporting information

Supporting Information

## Data Availability

Data are available through the request portal at the Center for Healthy Aging and Development Studies, Peking University.
