# Supplementary material for: Plant‐based dietary patterns and cognitive function: A prospective cohort analysis of elderly individuals in China (2008–2018)
Source: Brain Behav. 2022 Jul 14;12(8):e2670. doi: 10.1002/brb3.2670 (PMC9392533; doi:10.1002/brb3.2670)
Supplement: Supplementary file 1 — Supporting Information [file BRB3-12-e2670-s001.docx]

**Plant-based dietary patterns and cognitive function: a prospective cohort analysis of elderly individuals in China (2008-2018)**

**eFigure 1.** Flow chart of the included CLHLS participants

**eFigure 2.** Spearman correlation analysis among food groups

**eTable 1.** A comparison of baseline characteristics between the participants without follow-up and those with follow-up

**eTable 2.** Construction and scores of plant-based diet indices

**eTable 3**. Odds ratios (95% CI) of developing cognitive impairment by quartiles of plant-based diet indices among the participants with follow-up surveys and with normal cognition at baseline, using education-specific cut-off points for MMSE categorization (n=6136)

**eTable 4**. Odds ratios (95% CI) of developing cognitive impairment by quartiles of plant-based diet indices among the participants with follow-up surveys and with normal cognition at baseline, adjusted for time varying health behavior and health status (n=6136)

**eTable 5.** Odds ratios (95% CI) of developing cognitive impairment by quartiles of plant-based diet indices among the participants with follow-up surveys, regardless of baseline cognitive function (n=9206)

**eTable 6.** Odds ratios (95% CI) of developing cognitive impairment by quartiles of plant-based diet indices among the participants with follow-up surveys, and with normal cognition at baseline, stratified by APOE ε4 status (n=4023)

**eTable 7.** Odds ratios (95% CI) of cognitive impairment by quartiles of modified healthy plant-based diet index among the participants with follow-up surveys

**eTable 8.** Odds ratios (95% CI) of developing cognitive impairment by quartiles of plant-based diet indices among the participants with follow-up surveys, and with normal cognition at baseline (n=6136), using MMSE scores >=18 as the reference group

**eTable 9.** Association between individual food groups and development of cognitive impairment among the participants with follow-up surveys and with normal cognitive function at baseline (n=6136)

**eTable 10.** Odds ratios (95% CI) of cognitive impairment by intake frequency of healthy and less healthy food, animal food among the participants with follow-up surveys and normal cognition at baseline

**Supplemental methods**

**eFigure 1. Flow chart of the included CLHLS participants**

4023 participants have APOE ε4 data

Exclude 3070 participants with abnormal cognition at baseline

Exclude 8721 participants die or lost follow-up before 2^nd^ survey

6136 participants have normal cognition at baseline

9206 participants have >=1 follow-up survey

17,927 participants have a baseline survey

Exclude 1492 participants

- With missing values in diet, cognition, and covariates

- Aged <65 years

19,419 CLHLS participants

(2008-2018)

**eFigure 2. Spearman correlation analysis among food groups**

**
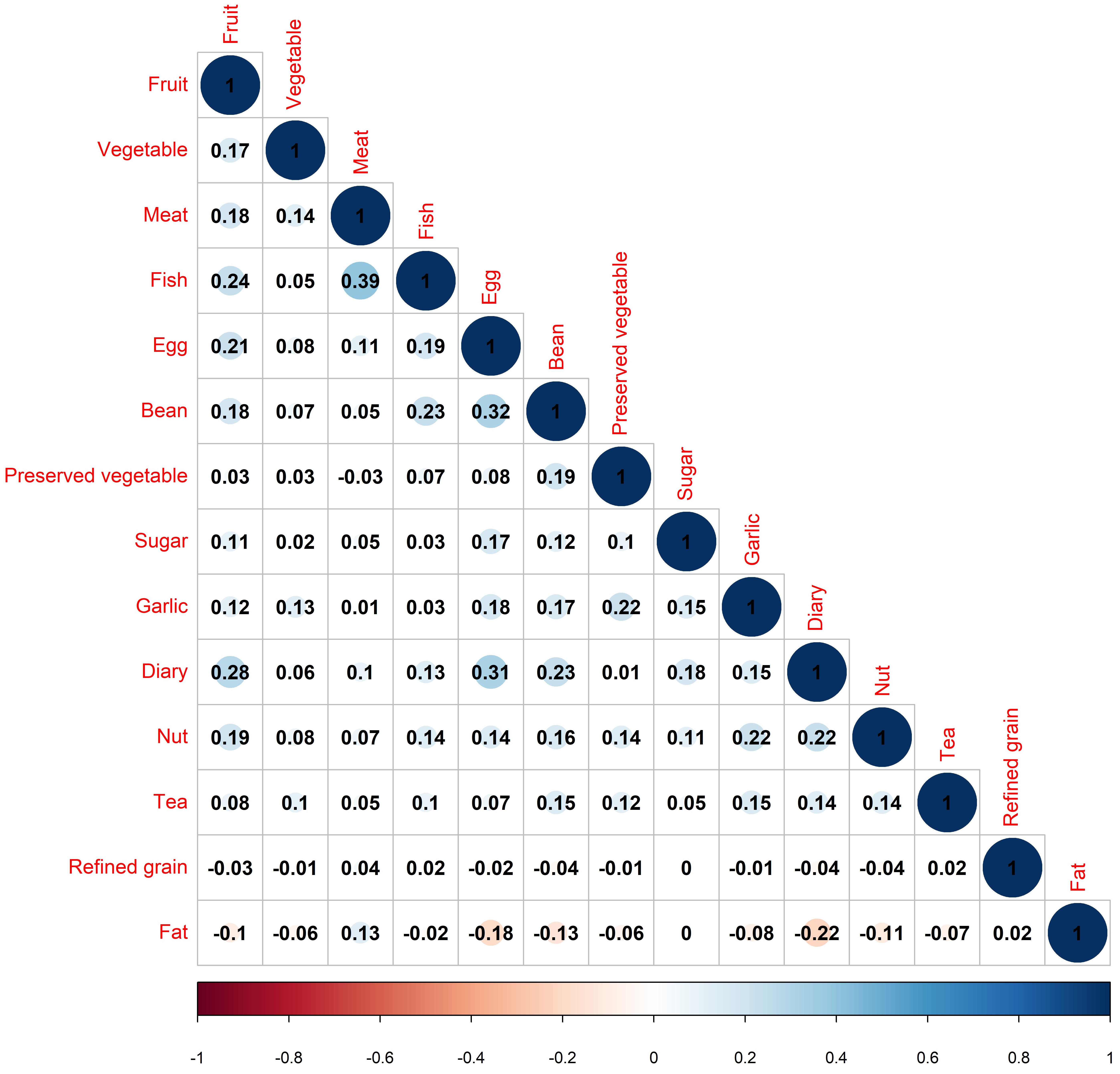
**

**eTable 1. A comparison of baseline characteristics between the participants without follow-up and those with follow-up**

| Characteristics | Without follow-up | With follow-up | p-value |
| --- | --- | --- | --- |
| N | 8721 | 9206 |  |
| Baseline PDI^†^ | 48.07±6.12 | 49.23±6.07 | <0.001 |
| Baseline hPDI^†^ | 46.01±5.66 | 46.72±5.60 | <0.001 |
| Baseline uPDI^†^ | 51.48±6.58 | 50.88±6.60 | <0.001 |
| Baseline MMSE | 17.78±10.86 | 23.92±8.09 | <0.001 |
| Age (years) ^†^ | 91.82±9.99 | 82.96±10.86 | <0.001 |
| Sex, males | 3462 (39.70) | 4190 (45.51) | <0.001 |
| Married | 1679 (19.25) | 3770 (40.95) | <0.001 |
| Rural residents | 6884 (78.94) | 7732 (83.99) | <0.001 |
| No formal education | 6035 (69.20) | 5310 (57.68) | <0.001 |
| Nonprofessional work | 8211 (94.15) | 8523 (92.58) | <0.001 |
| Financial dependent | 7105 (81.47) | 6383 (69.34) | <0.001 |
| Social and leisure activity index^†^ | 1.64±1.47 | 2.45±1.50 | <0.001 |
| Current smoker | 1223 (14.02) | 1826 (19.83) | <0.001 |
| Current drinker | 1230 (14.10) | 1783 (19.37) | <0.001 |
| Physical activity | 1869 (21.43) | 2880 (31.28) | <0.001 |
| Geographic region |  |  | <0.001 |
| Central China | 1679 (19.25) | 1724 (18.73) |  |
| Eastern China | 3149 (36.11) | 3450 (37.48) |  |
| Northeastern China | 656 (7.52) | 585 (6.35) |  |
| Northern China | 368 (4.22) | 349 (3.79) |  |
| Northwestern China | 59 (0.68) | 110 (1.19) |  |
| Southern China | 1715 (19.67) | 1953 (21.21) |  |
| Southwestern China | 1095 (12.56) | 1035 (11.24) |  |
| BMI (kg/m^2^)* | 19.86±3.53 | 20.90±5.90 | <0.001 |
| Daily vitamin A/C/E intake | 554 (6.35) | 496 (5.39) | <0.001 |
| Self-reported hypertension | 1594 (18.28) | 2017 (21.91) | <0.001 |
| Self-reported diabetes | 208 (2.39) | 246 (2.67) | <0.001 |
| Self-reported heart diseases | 758 (8.69) | 817 (8.87) | <0.001 |
| Self-reported cerebrovascular disease | 564 (6.47) | 520 (5.65) | <0.001 |
| Self-reported dyslipidemia | 124 (1.42) | 143 (1.55) | <0.001 |

^†^mean±SD was reported.

**eTable 2. Construction and scores of plant-based diet indices**

| Food category | Food | Frequency | PDI | hPDI | Modified hPDI | uPDI |
| --- | --- | --- | --- | --- | --- | --- |
| Healthy plant food | Whole grain | Yes | 5 | 5 | 5 | 1 |
|  |  | No | 1 | 1 | 1 | 5 |
|  | Vegetable oil | Yes | 5 | 5 | 5 | 1 |
|  |  | No | 1 | 1 | 1 | 5 |
|  | Fruit | Almost everyday | 5 | 5 | 5 | 1 |
|  |  | Quite often | 4 | 4 | 4 | 2 |
|  |  | Occasionally | 2 | 2 | 2 | 4 |
|  |  | Rarely or never | 1 | 1 | 1 | 5 |
|  | Vegetable | Almost everyday | 5 | 5 | 5 | 1 |
|  |  | Quite often | 4 | 4 | 4 | 2 |
|  |  | Occasionally | 2 | 2 | 2 | 4 |
|  |  | Rarely or never | 1 | 1 | 1 | 5 |
|  | Legume | Almost everyday | 5 | 5 | 5 | 1 |
|  |  | >=1 time/week | 4 | 4 | 4 | 2 |
|  |  | >=1 time/month | 3 | 3 | 3 | 3 |
|  |  | Occasionally | 2 | 2 | 2 | 4 |
|  |  | Rarely or never | 1 | 1 | 1 | 5 |
|  | Garlic | Almost everyday | 5 | 5 | 5 | 1 |
|  |  | >=1 time/week | 4 | 4 | 4 | 2 |
|  |  | >=1 time/month | 3 | 3 | 3 | 3 |
|  |  | Occasionally | 2 | 2 | 2 | 4 |
|  |  | Rarely or never | 1 | 1 | 1 | 5 |
|  | Nut | Almost everyday | 5 | 5 | 5 | 1 |
|  |  | >=1 time/week | 4 | 4 | 4 | 2 |
|  |  | >=1 time/month | 3 | 3 | 3 | 3 |
|  |  | Occasionally | 2 | 2 | 2 | 4 |
|  |  | Rarely or never | 1 | 1 | 1 | 5 |
|  | Tea | Almost everyday | 5 | 5 | 5 | 1 |
|  |  | >=1 time/week | 4 | 4 | 4 | 2 |
|  |  | >=1 time/month | 3 | 3 | 3 | 3 |
|  |  | Occasionally | 2 | 2 | 2 | 4 |
|  |  | Rarely or never | 1 | 1 | 1 | 5 |
| Less healthy plant food | Refined grain | Yes | 5 | 1 | 1 | 5 |
|  |  | No | 1 | 5 | 5 | 1 |
|  | Sugar | Almost everyday | 5 | 1 | 1 | 5 |
|  |  | >=1 time/week | 4 | 2 | 2 | 4 |
|  |  | >=1 time/month | 3 | 3 | 3 | 3 |
|  |  | Occasionally | 2 | 4 | 4 | 2 |
|  |  | Rarely or never | 1 | 5 | 5 | 1 |
|  | Salt-preserved vegetable | Almost everyday | 5 | 1 | 1 | 5 |
|  |  | >=1 time/week | 4 | 2 | 2 | 4 |
|  |  | >=1 time/month | 3 | 3 | 3 | 3 |
|  |  | Occasionally | 2 | 4 | 4 | 2 |
|  |  | Rarely or never | 1 | 5 | 5 | 1 |
| Animal food | Animal fat | Yes | 1 | 1 | 1 | 1 |
|  |  | No | 5 | 5 | 5 | 5 |
|  | Meat | Almost everyday | 1 | 1 | 1 | 1 |
|  |  | >=1 time/week | 2 | 2 | 2 | 2 |
|  |  | >=1 time/month | 3 | 3 | 3 | 3 |
|  |  | Occasionally | 4 | 4 | 4 | 4 |
|  |  | Rarely or never | 5 | 5 | 5 | 5 |
|  | Fish | Almost everyday | 1 | 1 | 5 | 1 |
|  |  | >=1 time/week | 2 | 2 | 4 | 2 |
|  |  | >=1 time/month | 3 | 3 | 3 | 3 |
|  |  | Occasionally | 4 | 4 | 2 | 4 |
|  |  | Rarely or never | 5 | 5 | 1 | 5 |
|  | Egg | Almost everyday | 1 | 1 | 1 | 1 |
|  |  | >=1 time/week | 2 | 2 | 2 | 2 |
|  |  | >=1 time/month | 3 | 3 | 3 | 3 |
|  |  | Occasionally | 4 | 4 | 4 | 4 |
|  |  | Rarely or never | 5 | 5 | 5 | 5 |
|  | Dairy products | Almost everyday | 1 | 1 | 1 | 1 |
|  |  | >=1 time/week | 2 | 2 | 2 | 2 |
|  |  | >=1 time/month | 3 | 3 | 3 | 3 |
|  |  | Occasionally | 4 | 4 | 4 | 4 |
|  |  | Rarely or never | 5 | 5 | 5 | 5 |

**eTable 3. Odds ratios (95% CI) of developing cognitive impairment by quartiles of plant-based diet indices among the participants with follow-up surveys and with normal cognition at baseline, using education-specific cut-off points for MMSE categorization (n=6136)**

| Diet indices | Model 1 | Model 2 | Model 3 |
| --- | --- | --- | --- |
| PDI |  |  |  |
| Quartile 1 | Ref | Ref | Ref |
| Quartile 2 | 0.80 (0.71, 0.92) | 0.79 (0.69, 0.91) | 0.80 (0.70, 0.91) |
| Quartile 3 | 0.54 (0.46, 0.62) | 0.54 (0.46, 0.63) | 0.54 (0.46, 0.63) |
| Quartile 4 | 0.34 (0.28, 0.40) | 0.34 (0.28, 0.42) | 0.34 (0.28, 0.42) |
| hPDI |  |  |  |
| Quartile 1 | Ref | Ref | Ref |
| Quartile 2 | 0.85 (0.75, 0.97) | 0.87 (0.76, 0.99) | 0.86 (0.75, 0.99) |
| Quartile 3 | 0.64 (0.55, 0.75) | 0.66 (0.56, 0.77) | 0.66 (0.57, 0.78) |
| Quartile 4 | 0.46 (0.39, 0.55) | 0.49 (0.41, 0.58) | 0.49 (0.41, 0.58) |
| uPDI |  |  |  |
| Quartile 1 | Ref | Ref | Ref |
| Quartile 2 | 1.46 (1.23, 1.74) | 1.38 (1.15, 1.65) | 1.38 (1.15, 1.65) |
| Quartile 3 | 1.91 (1.61, 2.27) | 1.73 (1.44, 2.08) | 1.72 (1.43, 2.08) |
| Quartile 4 | 2.67 (2.26, 3.15) | 2.34 (1.95, 2.81) | 2.33 (1.94, 2.81) |

Note:

Model 1 was adjusted for age.

Model 2 was multivariable-adjusted for age (years), sex (male or female), marital status (married or unmarried), urban/rural residence, education (with or without formal education), occupation before age 60 (professional or non-professional work), financial status (financial independence or dependence), social and leisure activity, smoking and drinking status (never, former or current smokers/drinkers), physical activity (yes or no), and geographic regions (Central China, Eastern China, Northeastern China, Northern China, Northwestern China, Southern China, and Southwestern China).

Model 3 was additionally adjusted for BMI (<18.5, 18.5-25.0, or >=25.0 kg/m^2^), vitamin A/C/E intake (almost everyday, >=1 time/week, >=1 time/month, occasionally, rarely or never) and status of five cardiometabolic diseases, including hypertension, diabetes, heart disease, cerebrovascular disease, and dyslipidemia (yes, no, or unknown).

**eTable 4. Odds ratios (95% CI) of developing cognitive impairment by quartiles of plant-based diet indices among the participants with follow-up surveys and with normal cognition at baseline, adjusted for time varying health behavior and health status (n=6136)**

| Diet indices | Model 1 | Model 2 | Model 3 |
| --- | --- | --- | --- |
| PDI |  |  |  |
| Quartile 1 | Ref | Ref | Ref |
| Quartile 2 | 0.91 (0.82, 1.00) | 0.99 (0.89, 1.11) | 0.98 (0.87, 1.09) |
| Quartile 3 | 0.64 (0.57, 0.71) | 0.78 (0.69, 0.88) | 0.77 (0.68, 0.87) |
| Quartile 4 | 0.43 (0.38, 0.49) | 0.60 (0.52, 0.69) | 0.61 (0.53, 0.71) |
| hPDI |  |  |  |
| Quartile 1 | Ref | Ref | Ref |
| Quartile 2 | 0.87 (0.79, 0.96) | 0.94 (0.84, 1.04) | 0.94 (0.84, 1.04) |
| Quartile 3 | 0.71 (0.64, 0.80) | 0.82 (0.72, 0.92) | 0.81 (0.71, 0.92) |
| Quartile 4 | 0.56 (0.50, 0.63) | 0.71 (0.62, 0.81) | 0.71 (0.62, 0.81) |
| uPDI |  |  |  |
| Quartile 1 | Ref | Ref | Ref |
| Quartile 2 | 1.32 (1.17, 1.49) | 1.06 (0.93, 1.20) | 1.05 (0.92, 1.20) |
| Quartile 3 | 1.74 (1.56, 1.95) | 1.18 (1.04, 1.33) | 1.18 (1.04, 1.35) |
| Quartile 4 | 2.56 (2.28, 2.88) | 1.45 (1.28, 1.66) | 1.49 (1.31, 1.71) |

Note:

Model 1 was adjusted for age.

Model 2 was multivariable-adjusted for age (years), sex (male or female), marital status (married or unmarried), urban/rural residence, education (with or without formal education), occupation before age 60 (professional or non-professional work), financial status (financial independence or dependence), social and leisure activity, smoking and drinking status (never, former or current smokers/drinkers), physical activity (yes or no), and geographic regions (Central China, Eastern China, Northeastern China, Northern China, Northwestern China, Southern China, and Southwestern China).

Model 3 was additionally adjusted for BMI (<18.5, 18.5-25.0, or >=25.0 kg/m^2^), vitamin A/C/E intake (almost everyday, >=1 time/week, >=1 time/month, occasionally, rarely or never) and status of five cardiometabolic diseases, including hypertension, diabetes, heart disease, cerebrovascular disease, and dyslipidemia (yes, no, or unknown).

**eTable 5. Odds ratios (95% CI) of developing cognitive impairment by quartiles of plant-based diet indices among the participants with follow-up surveys, regardless of baseline cognitive function (n=9206)**

| Diet indices | Model 1 | Model 2 | Model 3 |
| --- | --- | --- | --- |
| PDI |  |  |  |
| Quartile 1 | Ref | Ref | Ref |
| Quartile 2 | 0.94 (0.87, 1.02) | 0.94 (0.87, 1.03) | 0.95 ( 0.87, 1.03) |
| Quartile 3 | 0.83 (0.76, 0.90) | 0.82 (0.75, 0.90) | 0.83 (0.76, 0.90) |
| Quartile 4 | 0.66 (0.61, 0.72) | 0.68 (0.62, 0.75) | 0.69 (0.63, 0.76) |
| hPDI |  |  |  |
| Quartile 1 | Ref | Ref | Ref |
| Quartile 2 | 0.92 (0.85, 0.99) | 0.94 (0.86, 1.02) | 0.94 (0.87, 1.03) |
| Quartile 3 | 0.83 (0.77, 0.90) | 0.88 (0.81, 0.95) | 0.89 (0.82, 0.96) |
| Quartile 4 | 0.71 (0.65, 0.78) | 0.77 (0.71, 0.85) | 0.78 (0.71, 0.86) |
| uPDI |  |  |  |
| Quartile 1 | Ref | Ref | Ref |
| Quartile 2 | 1.43 (1.32, 1.56) | 1.23 (1.13, 1.34) | 1.22 (1.12, 1.33) |
| Quartile 3 | 1.86 (1.70, 2.03) | 1.48 (1.35, 1.63) | 1.47 (1.33, 1.61) |
| Quartile 4 | 2.67 (2.44, 2.91) | 1.95 (1.78, 2.15) | 1.93 (1.75, 2.12) |

Note:

Model 1 was adjusted for age.

Model 2 was multivariable-adjusted for age (years), sex (male or female), marital status (married or unmarried), urban/rural residence, education (with or without formal education), occupation before age 60 (professional or non-professional work), financial status (financial independence or dependence), social and leisure activity, smoking and drinking status (never, former or current smokers/drinkers), physical activity (yes or no), and geographic regions (Central China, Eastern China, Northeastern China, Northern China, Northwestern China, Southern China, and Southwestern China).

Model 3 was additionally adjusted for BMI (<18.5, 18.5-25.0, or >=25.0 kg/m^2^), vitamin A/C/E intake (almost everyday, >=1 time/week, >=1 time/month, occasionally, rarely or never) and status of five cardiometabolic diseases, including hypertension, diabetes, heart disease, cerebrovascular disease, and dyslipidemia (yes, no, or unknown).

**eTable 6. Odds ratios (95% CI) of developing cognitive impairment by quartiles of plant-based diet indices among the participants with follow-up surveys, and with normal cognition at baseline, stratified by APOE ε4 status (n=4023)**

| Diet indices | APOE non-ε4 carriers  (n=3285) | APOE ε4 carriers  (n=738) |
| --- | --- | --- |
| PDI |  |  |
| Quartile 1 | Ref | Ref |
| Quartile 2 | 0.86 (0.74, 1.00) | 0.75 (0.54, 1.03) |
| Quartile 3 | 0.59 (0.50, 0.70) | 0.68 (0.48, 0.95) |
| Quartile 4 | 0.41 (0.34, 0.51) | 0.44 (0.30, 0.66) |
| hPDI |  |  |
| Quartile 1 | Ref | Ref |
| Quartile 2 | 0.85 (0.74, 0.99) | 1.05 (0.78, 1.42) |
| Quartile 3 | 0.76 (0.64, 0.90) | 0.83 (0.58, 1.17) |
| Quartile 4 | 0.60 (0.50, 0.73) | 0.68 (0.47, 1.00) |
| uPDI |  |  |
| Quartile 1 | Ref | Ref |
| Quartile 2 | 1.24 (1.04, 1.48) | 1.18 (0.80, 1.74) |
| Quartile 3 | 1.60 (1.35, 1.89) | 1.43 (1.00, 2.07) |
| Quartile 4 | 2.06 (1.71, 2.47) | 2.11 (1.45, 3.05) |

Note:

The regression models were multivariable-adjusted for age (years), sex (male or female), marital status (married or unmarried), urban/rural residence, education (with or without formal education), occupation before age 60 (professional or non-professional work), financial status (financial independence or dependence), social and leisure activity, smoking and drinking status (never, former or current smokers/drinkers), physical activity (yes or no), and geographic regions (Central China, Eastern China, Northeastern China, Northern China, Northwestern China, Southern China, and Southwestern China).

**eTable 7. Odds ratios (95% CI) of cognitive impairment by quartiles of modified healthy plant-based diet index among the participants with follow-up surveys**

| **Among the participants with follow-up surveys (n=9206)** | | | |
| --- | --- | --- | --- |
| Diet indices | Model 1 | Model 2 | Model 3 |
| Modified hPDI |  |  |  |
| Quartile 1 | Ref | Ref | Ref |
| Quartile 2 | 0.84 (0.78, 0.91) | 0.87 (0.81, 0.95) | 0.87 (0.80, 0.95) |
| Quartile 3 | 0.69 (0.64, 0.75) | 0.75 (0.69, 0.81) | 0.76 (0.70, 0.82) |
| Quartile 4 | 0.51 (0.46, 0.55) | 0.62 (0.56, 0.68) | 0.62 (0.57, 0.69) |
| **Among the participants with follow-up surveys and with normal cognition at baseline (n=6136)** | | | |
| Diet indices | Model 1 | Model 2 | Model 3 |
| Modified hPDI |  |  |  |
| Quartile 1 | Ref | Ref | Ref |
| Quartile 2 | 0.78 (0.71, 0.86) | 0.80 (0.72, 0.89) | 0.80 (0.72, 0.89) |
| Quartile 3 | 0.56 (0.50, 0.63) | 0.60 (0.53, 0.68) | 0.60 (0.53, 0.68) |
| Quartile 4 | 0.45 (0.40, 0.50) | 0.52 (0.45, 0.59) | 0.52 (0.46, 0.59) |

Note:

Model 1 was adjusted for age.

Model 2 was multivariable-adjusted for age (years), sex (male or female), marital status (married or unmarried), urban/rural residence, education (with or without formal education), occupation before age 60 (professional or non-professional work), financial status (financial independence or dependence), social and leisure activity, smoking and drinking status (never, former or current smokers/drinkers), physical activity (yes or no), and geographic regions (Central China, Eastern China, Northeastern China, Northern China, Northwestern China, Southern China, Southwestern China).

Model 3 was additionally adjusted for BMI (<18.5, 18.5-25.0, or >=25.0 kg/m^2^), vitamin A/C/E intake (almost everyday, >=1 time/week, >=1 time/month, occasionally, rarely or never) and status of five cardiometabolic diseases, including hypertension, diabetes, heart disease, cerebrovascular disease, and dyslipidemia (yes, no, or unknown).

**eTable 8. Odds ratios (95% CI) of developing cognitive impairment by quartiles of plant-based diet indices among the participants with follow-up surveys, and with normal cognition at baseline (n=6136), using MMSE scores >=18 as the reference group**

| Diet indices | Model 1 | Model 2 | Model 3 |
| --- | --- | --- | --- |
| PDI |  |  |  |
| Quartile 1 | Ref | Ref | Ref |
| Quartile 2 | 0.82 (0.72, 0.94) | 0.79 (0.69, 0.91) | 0.80 (0.69, 0.91) |
| Quartile 3 | 0.55 (0.48, 0.64) | 0.54 (0.46, 0.63) | 0.54 (0.46, 0.64) |
| Quartile 4 | 0.32 (0.26, 0.39) | 0.32 (0.26, 0.39) | 0.32 (0.26, 0.39) |
| hPDI |  |  |  |
| Quartile 1 | Ref | Ref | Ref |
| Quartile 2 | 0.85 (0.75, 0.97) | 0.86 (0.75, 0.98) | 0.86 (0.75, 0.98) |
| Quartile 3 | 0.66 (0.57, 0.78) | 0.69 (0.58, 0.80) | 0.69 (0.59, 0.81) |
| Quartile 4 | 0.44 (0.37, 0.52) | 0.46 (0.39, 0.55) | 0.47 (0.39, 0.56) |
| uPDI |  |  |  |
| Quartile 1 | Ref | Ref | Ref |
| Quartile 2 | 1.35 (1.13, 1.61) | 1.23 (1.03, 1.47) | 1.22 (1.02, 1.47) |
| Quartile 3 | 1.89 (1.61, 2.22) | 1.65 (1.39, 1.95) | 1.65 (1.39, 1.96) |
| Quartile 4 | 2.90 (2.47, 3.40) | 2.40 (2.02, 2.86) | 2.39 (2.00, 2.86) |

Note:

Model 1 was adjusted for age.

Model 2 was multivariable-adjusted for age (years), sex (male or female), marital status (married or unmarried), urban/rural residence, education (with or without formal education), occupation before age 60 (professional or non-professional work), financial status (financial independence or dependence), social and leisure activity, smoking and drinking status (never, former or current smokers and drinkers), physical activity (yes or no), and geographic regions (Central China, Eastern China, Northeastern China, Northern China, Northwestern China, Southern China, Southwestern China).

Model 3 was additionally adjusted for BMI (<18.5, 18.5-25.0, or >=25.0 kg/m^2^), vitamin A/C/E intake (almost everyday, >=1 time/week, >=1 time/month, occasionally, rarely or never) and status of five cardiometabolic diseases, including hypertension, diabetes, heart disease, cerebrovascular disease, and dyslipidemia (yes, no, or unknown).

**eTable 9. Association between individual food groups and development of cognitive impairment among the participants with follow-up surveys and with normal cognitive function at baseline (n=6136)**

| Food group | Baseline consumption frequency | OR (95% CI) |
| --- | --- | --- |
| Whole grain |  |  |
| Yes | 190 (3.10) | 0.90 (0.70, 1.15) |
| No | 5946 (96.90) | Ref |
| Vegetable oil |  |  |
| Yes | 5170 (84.26) | 1.20 (1.04, 1.38) |
| No | 966 (15.74) | Ref |
| Fruit |  |  |
| Almost everyday | 870 (14.18) | 0.66 (0.56, 0.77) |
| Quite often | 1846 (30.09) | 0.52 (0.46, 0.59) |
| Occasionally | 2264 (36.89) | 0.65 (0.58, 0.73) |
| Rarely or never | 1156 (18.84) | Ref |
| Vegetable |  |  |
| Almost everyday | 4068 (66.30) | 0.42 (0.33, 0.53) |
| Quite often | 1622 (26.43) | 0.51 (0.40, 0.65) |
| Occasionally | 369 (6.01) | 0.76 (0.58, 0.99) |
| Rarely or never | 77 (1.25) | Ref |
| Legume |  |  |
| Almost everyday | 1011 (16.48) | 0.87 (0.73, 1.03) |
| >=1 time/week | 2392 (38.97) | 1.04 (0.90, 1.20) |
| >=1 time/month | 1059 (17.26) | 1.13 (0.96, 1.31) |
| Occasionally | 1009 (16.44) | 0.94 (0.79, 1.10) |
| Rarely or never | 665 (10.84) | Ref |
| Garlic |  |  |
| Almost everyday | 1289 (21.01) | 0.86 (0.75, 0.99) |
| >=1 time/week | 1381 (22.51) | 0.88 (0.78, 1.00) |
| >=1 time/month | 730 (11.90) | 0.88 (0.76, 1.02) |
| Occasionally | 1369 (22.31) | 0.85 (0.74, 0.96) |
| Rarely or never | 1367 (22.28) | Ref |
| Nut |  |  |
| Almost everyday | 248 (4.04) | 0.71 (0.54, 0.93) |
| >=1 time/week | 622 (10.14) | 0.76 (0.65, 0.90) |
| >=1 time/month | 606 (9.88) | 0.74 (0.62, 0.87) |
| Occasionally | 1448 (23.60) | 0.78 (0.69, 0.88) |
| Rarely or never | 3212 (52.34) | Ref |
| Tea |  |  |
| Almost everyday | 2133 (34.76) | 0.55 (0.49, 0.62) |
| >=1 time/week | 361 (5.88) | 0.88 (0.74, 1.06) |
| >=1 time/month | 145 (2.36) | 1.01 (0.78, 1.31) |
| Occasionally | 480 (7.82) | 0.66 (0.54, 0.80) |
| Rarely or never | 3017 (49.17) | Ref |
| Food group | Baseline consumption frequency | OR (95% CI) |
| Salt preserved vegetable |  |  |
| Almost everyday | 1306 (21.28) | 0.74 (0.65, 0.85) |
| >=1 time/week | 1078 (17.57) | 1.04 (0.92, 1.18) |
| >=1 time/month | 619 (10.09) | 0.96 (0.83, 1.11) |
| Occasionally | 1051 (17.13) | 0.85 (0.75, 0.97) |
| Rarely or never | 2082 (33.91) | Ref |
| Sugar |  |  |
| Almost everyday | 846 (13.79) | 1.04 (0.91, 1.19) |
| >=1 time/week | 1199 (19.54) | 0.95 (0.84, 1.08) |
| >=1 time/month | 736 (11.99) | 0.91 (0.78, 1.05) |
| Occasionally | 1388 (22.62) | 0.82 (0.73, 0.93) |
| Rarely or never | 1967 (32.06) | Ref |
| Meat |  |  |
| Almost everyday | 1900 (30.96) | 1.32 (1.10, 1.59) |
| >=1 time/week | 2495 (40.66) | 1.09 (0.92, 1.30) |
| >=1 time/month | 685 (11.16) | 1.17 (0.96, 1.43) |
| Occasionally | 555 (9.04) | 0.89 (0.71, 1.10) |
| Rarely or never | 501 (8.16) | Ref |
| Fish |  |  |
| Almost everyday | 485 (7.90) | 0.73 (0.59, 0.89) |
| >=1 time/week | 2256 (36.77) | 0.79 (0.69, 0.90) |
| >=1 time/month | 1276 (20.80) | 0.80 (0.70, 0.93) |
| Occasionally | 1045 (17.03) | 0.70 (0.61, 0.82) |
| Rarely or never | 1074 (17.50) | Ref |
| Egg |  |  |
| Almost everyday | 2090 (34.07) | 0.86 (0.73, 1.01) |
| >=1 time/week | 2219 (36.15) | 1.00 (0.86, 1.17) |
| >=1 time/month | 761 (12.40) | 1.03 (0.86, 1.23) |
| Occasionally | 551 (8.98) | 1.06 (0.87, 1.29) |
| Rarely or never | 515 (8.39) | Ref |
| Dairy products |  |  |
| Almost everyday | 1044 (17.01) | 1.16 (1.01, 1.33) |
| >=1 time/week | 700 (11.41) | 1.31 (1.14, 1.50) |
| >=1 time/month | 422 (6.88) | 1.19 (1.01, 1.40) |
| Occasionally | 903 (14.72) | 1.14 (0.99, 1.30) |
| Rarely or never | 3067 (49.98) | Ref |

Note: The regression models were multivariable-adjusted for age (years), sex (male or female), marital status (married or unmarried), urban/rural residence, education (with or without formal education), occupation before age 60 (professional or non-professional work), financial status (financial independence or dependence), social and leisure activity, smoking and drinking status (never, former or current smokers/drinkers), physical activity (yes or no), and geographic regions (Central China, Eastern China, Northeastern China, Northern China, Northwestern China, Southern China, and Southwestern China).

**eTable 10. Odds ratios (95% CI) of cognitive impairment by intake frequency of healthy and less healthy food, animal food among the participants with follow-up surveys and normal cognition at baseline**

| Intake frequency | Healthy plant food | Less healthy plant food | Animal food |
| --- | --- | --- | --- |
| Quartile 1 | Ref | Ref | Ref |
| Quartile 2 | 0.75 (0.67, 0.83) | 0.96 (0.86, 1.07) | 1.03 (0.93, 1.14) |
| Quartile 3 | 0.53 (0.48, 0.60) | 0.98 (0.88, 1.10) | 0.89 (0.79, 1.01) |
| Quartile 4 | 0.34 (0.29, 0.40) | 0.93 (0.81, 1.06) | 0.87 (0.77, 0.99) |

Note: Quartile 1 indicated least frequent intake while quartile 4 indicated more frequent intake.

The regression models were multivariable-adjusted for age (years), sex (male or female), marital status (married or unmarried), urban/rural residence, education (with or without formal education), occupation before age 60 (professional or non-professional work), financial status (financial independence or dependence), social and leisure activity, smoking and drinking status (never, former or current smokers/drinkers), physical activity (yes or no), and geographic regions (Central China, Eastern China, Northeastern China, Northern China, Northwestern China, Southern China, and Southwestern China).

**Supplemental methods**

**Dietary assessment**

We divided 16 food groups into three food groups. Healthy plant food groups included whole grains, fruits, fresh vegetables, legumes, garlic, vegetable oils, nut, and tea, whereas less healthy plant food groups included refined grains, salt-preserved vegetables, and sugar. Animal food groups included animal fat, eggs, fish, meat, and dairy products. Dietary assessment categorization was decided a priori.

The CLHLS recorded the intake frequency as “almost everyday” or “>=1 time per week” or “>=1 time per month” or “occasionally” or “rarely or never” for most food groups, including legumes, garlic, nut, tea, salt-preserved vegetables, sugar, eggs, fish, meat, and dairy products. They were scored 1, 2, 3, 4, and 5. The CLHLS recorded the intake frequency of fruits and fresh vegetables as “almost everyday” or “quite often” or “occasionally” or “rarely or never”, which were scored 1, 2, 4, and 5. Based on the information of staple food and cooking oil, we defined whole grains (corn), refined grains (rice and flour), vegetable oil, and animal fats, which were coded as yes and no, and scored 1 and 5, depending on indices. For PDI, we scored 5 for the most frequent consumption, and 1 for the least frequent consumption of healthy and less healthy plant food groups (positive scores). We scored 1 for the most frequent consumption, and 5 for the least frequent consumption of animal food groups (reverse scores). We scored 5 for the consumption of whole grain, vegetable oil, and refined grain, and scored 1 for the consumption of animal fat. For hPDI, we assigned positive scores to healthy plant food groups, and reverse scores to less healthy plant food and animal food groups. We scored 5 for the consumption of whole grain and vegetable oil, and scored 1 for the consumption of refined grain and animal fat. For uPDI, we gave positive scores to less healthy plant food groups, and reverse scores to healthy plant and animal food groups. We scored 1 for the consumption of whole grain, vegetable oil, and animal fat, and scored 5 for the consumption of refined grain. In addition, observational studies showed omega-3 fatty acids, common in fatty fish, were associated with lower risks of cognitive decline. We constructed a modified hPDI, which is similar to hPDI except giving positive scores for the consumption of fish. More details on constructing and scoring PDI, hPDI, modified hPDI, and uPDI could be found in eTable 2.

We summed the scores for 16 included food groups to obtain the indices, which ranges from 16 to 80 theoretically. Higher PDI indicated more frequent consumption of plant food groups; higher hPDI indicated more frequent consumption of healthy plant food groups; while higher uPDI indicated more frequent consumption of less healthy plant food groups. We divided the indices into the quartile for statistical analysis.
